# Triggering of suicidal erythrocyte death by uremic toxin indoxyl sulfate

**DOI:** 10.1186/1471-2369-14-244

**Published:** 2013-11-04

**Authors:** Mohamed Siyabeldin E Ahmed, Majed Abed, Jakob Voelkl, Florian Lang

**Affiliations:** 1 Department of Physiology, University of Tuebingen, Gmelinstraße 5, 72076 Tuebingen, Germany

**Keywords:** Phosphatidylserine, Indoxyl sulfate, Calcium, Cell volume, Eryptosis

## Abstract

**Background:**

Anemia in end stage renal disease is attributed to impaired erythrocyte formation due to erythropoietin and iron deficiency. On the other hand, end stage renal disease enhances eryptosis, the suicidal erythrocyte death characterized by cell shrinkage and phosphatidylserine-exposure at the erythrocyte surface. Eryptosis may be triggered by increase of cytosolic Ca^2+^-activity ([Ca^2+^]_i_) and by ceramide, which sensitizes erythrocytes to [Ca^2+^]_i_. Mechanisms triggering eryptosis in endstage renal disease remained enigmatic. The present study explored the effect of indoxyl sulfate, an uremic toxin accumulated in blood of patients with chronic kidney disease.

**Methods:**

Cell volume was estimated from forward scatter, phosphatidylserine-exposure from annexin V binding, ceramide abundance by specific antibodies, hemolysis from hemoglobin release, and [Ca^2+^]_i_ from Fluo3-fluorescence.

**Results:**

A 48 hours exposure to indoxyl sulfate significantly increased [Ca^2+^]_i_ (≥ 300 μM), significantly decreased forward scatter (≥ 300 μM) and significantly increased annexin-V-binding (≥ 50 μM). Indoxyl sulfate (150 μM) induced annexin-V-binding was virtually abolished in the nominal absence of extracellular Ca^2+^. Indoxyl sulfate (150 μM) further enhanced ceramide abundance.

**Conclusion:**

Indoxyl sulfate stimulates suicidal erythrocyte death or eryptosis, an effect in large part due to stimulation of extracellular Ca^2+^entry with subsequent stimulation of cell shrinkage and cell membrane scrambling.

## Background

Severe complications of end stage renal disease include anemia [1,2], which is at least in part the result of restricted renal erythropoietin release and subsequent impairment of erythropoiesis [3,4]. In end stage renal disease erythropoiesis is typically further compromized by iron deficiency [5,6]. In addition, compelling evidence points to accelerated clearance of circulating erythrocytes in end stage renal disease [7]. The accelerated clearance of erythrocytes in end stage renal disease may at least partially be due to enhanced eryptosis, a suicidal death of erythrocytes characterized by cell shrinkage and cell membrane scrambling with phosphatidylserine exposure at the erythrocyte surface [8,9]. As a matter of fact, the concentration of phosphatidylserine exposing erythrocytes has been found to be twice as high in patients on dialysis than in the common population [10]. As phosphatidylserine exposing erythrocytes are rapidly cleared from circulating blood *in vivo*[9], a doubling of phosphatiylserine exposing erythrocytes in circulating blood is expected to reflect a decrease of erythrocyte life span to half. As long as erythrocyte formation is not enhanced, a decrease of erythrocyte life span would lead to a quantitatively similar decrease of erythrocyte count in circulating blood. Thus, the contribution of eryptosis to anemia in CKD patients is probably substantial.

The most important trigger of eryptosis is enhanced cytosolic Ca^2+^ concentration ([Ca^2+^]_i_) [8,9]. The increase of [Ca^2+^]_i_ may result from Ca^2+^ entry through Ca^2+^-permeable cation channels [9], which are activated by oxidation [9]. Increased [Ca^2+^]_i_ leads to eryptotic cell shrinkage by activation of Ca^2+^-sensitive K^+^ channels [9], K^+^ exit, hyperpolarization, Cl^-^ exit and thus cellular KCl and water loss [9]. Increased [Ca^2+^]_i_ triggers phosphatidylserine exposure at the cell surface by triggering cell membrane scrambling [9]. Eryptosis may further be stimulated by ceramide [9], energy depletion [9], caspase activation [9,11,12] and deranged activity of kinases such as protein kinase C [9], AMP activated kinase AMPK [9], cGMP-dependent protein kinase [9], Janus-activated kinase JAK3 [13], casein kinase [14,15], p38 kinase [16], as well as sorafenib [17] and sunitinib [18] sensitive kinases. Eryptosis is further triggered by a wide variety of xenobiotics and is enhanced in a variety of clinical disorders [9,19-37].

Little is known about mechanisms underlying enhanced eryptosis in endstage renal disease. At least in theory, eryptosis may be stimulated by some uremic toxins. As a matter of fact, eryptosis has previously been shown to be triggered by the uremic toxins vanadate [9], acrolein [38] and methylglyoxal [9]. A further uremic toxins that could contribute to anemia in chronic kidney disease is indoxyl sulfate [39,40], which is at least partially effective by suppression of erythropoietin production [41]. Further effects of indoxyl sulfate include downregulation of Klotho [42], induction of oxidative stress [42,43], up-regulation of NFκB [42], aortic calcification and aortic wall thickening [42], interference with wound repair [44], triggering of cell senescence [42], stimulation of cardiac and renal fibrosis [42,45] and acceleration of renal disease progression [42]. Indoxyl sulfate is generated by colonic microbes [46] and accumulates in blood, if renal excretion is impaired [42]. Indoxyl sulfate induces apoptosis, the suicidal death of nucleated cells, an effect involving ERK1/2 and p38 MAP kinase [47].

The present study explored, whether eryptosis is stimulated by indoxyl sulfate. To this end, the effect of indoxyl sulfate on [Ca^2+^]_i_, cell volume, ceramide formation and phosphatidylserine abundance at the erythrocyte surface were determined.

## Methods

### Erythrocytes, solutions and chemicals

Leukocyte-depleted erythrocytes were kindly provided by the blood bank of the University of Tübingen. The study is approved by the ethics committee of the University of Tübingen (184/2003 V). Erythrocytes were incubated *in vitro* at a hematocrit of 0.4% in Ringer solution containing (in mM) 125 NaCl, 5 KCl, 1 MgSO_4_, 32 N-2-hydroxyethylpiperazine-N-2-ethanesulfonic acid (HEPES), 5 glucose, 1 CaCl_2_; pH 7.4 at 37°C for 48 h. Where indicated, erythrocytes were exposed to indoxyl sulfate potassium salt (Sigma-Aldrich, Steinheim, Germany) at the indicated concentrations. In Ca^2+^-free Ringer solution, 1 mM CaCl_2_ was substituted by 1 mM glycol-bis(2-aminoethylether)-N,N,N’,N’-tetraacetic acid (EGTA).

### FACS analysis of annexin-V-binding and forward scatter

After incubation under the respective experimental condition, 50 μl cell suspension was washed in Ringer solution containing 5 mM CaCl_2_ and then stained with Annexin-V-FITC (1:200 dilution; ImmunoTools, Friesoythe, Germany) in this solution at 37°C for 20 min under protection from light. In the following, the forward scatter (FSC) of the cells was determined, and annexin-V fluorescence intensity was measured with an excitation wavelength of 488 nm and an emission wavelength of 530 nm on a FACS Calibur (BD, Heidelberg, Germany).

### Measurement of intracellular Ca^2+^

After incubation erythrocytes were washed in Ringer solution and then loaded with Fluo-3/AM (Biotium, Hayward, USA) in Ringer solution containing 5 mM CaCl_2_ and 2 μM Fluo-3/AM. The cells were incubated at 37°C for 30 min and washed twice in Ringer solution containing 5 mM CaCl_2_. The Fluo-3/AM-loaded erythrocytes were resuspended in 200 μl Ringer. Then, Ca^2+^-dependent fluorescence intensity was measured with an excitation wavelength of 488 nm and an emission wavelength of 530 nm on a FACS Calibur.

### Measurement of hemolysis

For the determination of hemolysis the samples were centrifuged (3 min at 400 g, room temperature) after incubation, and the supernatants were harvested. As a measure of hemolysis, the hemoglobin (Hb) concentration of the supernatant was determined photometrically at 405 nm. The absorption of the supernatant of erythrocytes lysed in distilled water was defined as 100% hemolysis.

### Determination of ceramide formation

For the determination of ceramide, a monoclonal antibody-based assay was used. After incubation, cells were stained for 1 hour at 37°C with 1 μg/ml anti-ceramide antibody (clone MID 15B4, Alexis, Grünberg, Germany) in PBS containing 0.1% bovine serum albumin (BSA) at a dilution of 1:5. The samples were washed twice with PBS-BSA. Subsequently, the cells were stained for 30 minutes with polyclonal fluorescein-isothiocyanate (FITC)-conjugated goat anti-mouse IgG and IgM specific antibody (Pharmingen, Hamburg, Germany) diluted 1:50 in PBS-BSA. Unbound secondary antibody was removed by repeated washing with PBS-BSA. The samples were then analyzed by flow cytometric analysis with an excitation wavelength of 488 nm and an emission wavelength of 530 nm.

### Statistics

Data are expressed as arithmetic means ± SEM. As indicated in the figure legends, statistical analysis was made using ANOVA and *t* test as appropriate. N denotes the number of different erythrocyte specimens studied. Since different erythrocyte specimens used in distinct experiments are differently susceptible to triggers of eryptosis, the same erythrocyte specimens have been used for control and experimental conditions.

## Results and discussion

**Figure 1:**
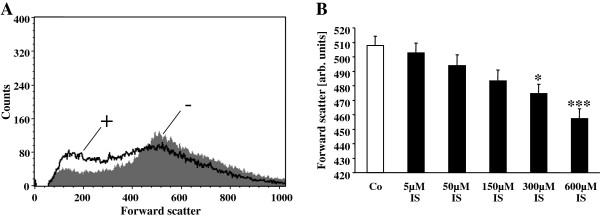
**Effect of indoxyl sulfate on erythrocyte forward scatter. A**. Original histogram of forward scatter of erythrocytes following exposure for 48 hours to Ringer solution without (-, grey) and with (+, black) presence of 600 μM indoxyl sulfate. **B**. Arithmetic means ± SEM (n = 24–25) of the normalized erythrocyte forward scatter (FSC) following incubation for 48 hours to Ringer solution without (white bar) or with (black bars) indoxyl sulfate (5–600 μM). *,*** (p < 0.05, 0.001) indicate significant difference from the absence of indoxyl sulfate (ANOVA).

The present study aimed to test whether indoxyl sulfate exposure triggers eryptosis, the suicidal erythrocyte death, which is characterized by cell shrinkage and by cell membrane scrambling. Cell volume was determined utilizing flow cytometry. As shown in Figure 1, a 48 hours treatment with indoxyl sulfate led to a decrease of forward scatter, an effect reaching statistical significance at 300 μM indoxyl sulfate concentration. Accordingly, indoxyl sulfate treatment was followed by erythrocyte shrinkage.

**Figure 2:**
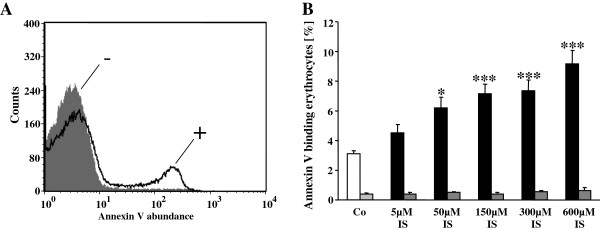
**Effect of indoxyl sulfate on phosphatidylserine exposure and hemolysis. A**. Original histogram of annexin-V-binding of erythrocytes following exposure for 48 hours to Ringer solution without (-, grey) and with (+, black) presence of 600 μM indoxyl sulfate. **B**. Arithmetic means ± SEM (n = 24–25) of erythrocyte annexin-V-binding following incubation for 48 hours to Ringer solution without (white bar) or with (black bars) presence of indoxyl sulfate (5–600 μM). For comparison, arithmetic means ± SEM (n = 5) of the percentage of hemolysis is shown as grey bars. *,*** (p < 0.05, 0.001) indicates significant difference from the absence of indoxyl sulfate for the respective measurements (ANOVA).

In order to analyze cell membrane scrambling, phosphatidylserine exposing erythrocytes were identified by annexin-V-binding in FACS analysis. As shown in Figure 2, a 48 hours treatment with indoxyl sulfate dose dependently increased the percentage of annexin-V-binding erythrocytes. This effect reached statistical significance at 50 μM indoxyl sulfate concentration. Accordingly, indoxyl sulfate stimulated erythrocyte cell membrane scrambling leading to phosphatidylserine exposure at the cell surface.

To explore, whether indoxyl sulfate exposure leads to hemolysis, the percentage of hemolysed erythrocytes was quantified by determination of hemoglobin abundance in the supernatant. As shown in Figure 2, treatment of erythrocytes for 48 hours with indoxyl sulfate did not significantly increase the hemoglobin concentration in the supernatant. (Figure 2). Thus, indoxyl sulfate triggered phosphatidylserine translocation at the cell membrane without appreciably permeabilizing the cell membrane to hemoglobin.

**Figure 3:**
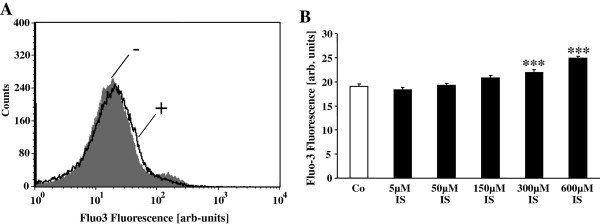
**Effect of indoxyl sulfate on erythrocyte cytosolic Ca**^**2+ **^**concentration. A**. Original histogram of Fluo3 fluorescence in erythrocytes following exposure for 48 hours to Ringer solution without (-, grey) and with (+, black) presence of 600 μM indoxyl sulfate. **B**. Arithmetic means ± SEM (n = 20) of the Fluo3 fluorescence (arbitrary units) in erythrocytes exposed for 48 hours to Ringer solution without (white bar) or with (black bars) indoxyl sulfate (5–600 μM).

Both, cell shrinkage and cell membrane scrambling are known to be triggered by an increase of cytosolic Ca^2+^ concentration ([Ca^2+^]_i_). Further experiments were thus performed to elucidate the effect of indoxyl sulfate on [Ca^2+^]_i_. Erythrocytes were exposed to Ringer solution in the absence or presence of indoxyl sulfate (5–600 μM). The erythrocytes were subsequently loaded with Fluo3-AM and Fluo3 fluorescence determined in FACS analysis. As shown in Figure 3, a 48 hours exposure of human erythrocytes to indoxyl sulfate was followed by an increase of Fluo3 fluorescence, an effect reaching statistical significance at 300 μM indoxyl sulfate concentration. Accordingly, indoxyl sulfate increased cytosolic Ca^2+^ concentration.

**Figure 4:**
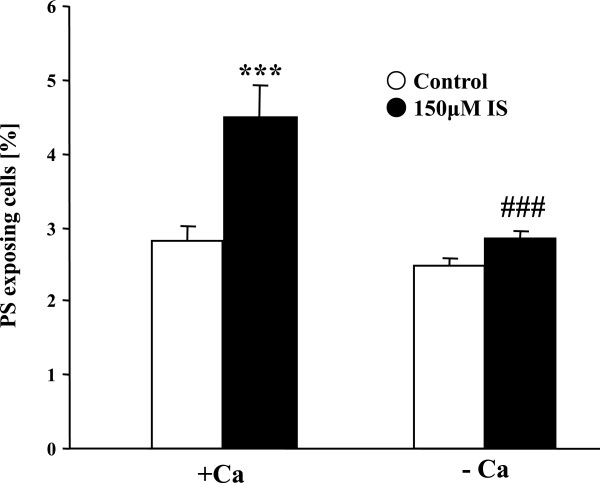
**Effect of Ca**^**2+ **^**withdrawal on indoxyl sulfate-induced annexin-V-binding.** Arithmetic means ± SEM (n = 10) of the percentage of annexin-V-binding erythrocytes after a 48 hours treatment with Ringer solution without (white bar) or with (black bars) 150 μM indoxyl sulfate in the presence (left bars, + Ca) and absence (right bars, - Ca) of calcium. *** (<0.001) indicates significant difference from respective control (absence of indoxyl sulfate) (ANOVA) ### (p < 0.001) indicates significant difference from the respective values in the presence of Ca^2+^.

In order to determine, whether the stimulation of cell membrane scrambling by indoxyl sulfate was secondary to an increase of [Ca^2+^]_i_, erythrocytes were exposed to 150 μM indoxyl sulfate for 48 hours either in the presence of extracellular Ca^2+^ (1 mM) or in the nominal absence of Ca^2+^ and presence of the Ca^2+^ chelator EGTA (1 mM). As shown in Figure 4, the effect of indoxyl sulfate on annexin-V-binding was virtually abolished in the nominal absence of extracellular Ca^2+^.

**Figure 5:**
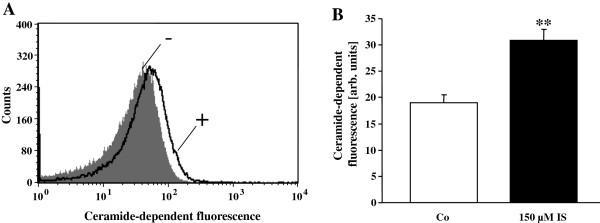
**Effect of indoxyl sulfate on ceramide formation. A**. Original histogram of anti-ceramide FITC-fluorescence in erythrocytes following exposure for 48 hours to Ringer solution without (-, grey) and with (+, black) presence of 150 μM indoxyl sulfate. **B**. Arithmetic means ± SEM (n = 5) of ceramide abundance after a 48 hours incubation in Ringer solution without (white bar) or with (black bars) indoxyl sulfate (150 μM). ** (p <0.01) indicates significant difference from control (absence of indoxyl sulfate) (t test).

A further series of experiments was performed to define the effect of indoxyl sulfate on formation of ceramide. Ceramide abundance at the cell surface was elucidated utilizing FITC-labeled anti-ceramide antibodies. As shown in Figure 5, treatment of erythrocytes with 150 μM indoxyl sulfate significantly increased ceramide abundance at the erythrocyte surface.

The present study uncovers a novel effect of indoxyl sulfate, i.e. the triggering of erythrocyte shrinkage and erythrocyte cell membrane scrambling, both hallmarks of suicidal erythrocyte death or eryptosis. The concentrations of indoxyl sulfate required for statistically significant stimulation of eryptosis were in the range of those encountered in uremic plasma [39,48].

The observed erythrocyte shrinkage following indoxyl sulfate treatment presumably resulted from increase of cytosolic Ca^2+^ concentration with subsequent activation of Ca^2+^ sensitive K^+^ channels [9,49], K^+^ exit, cell membrane hyperpolarisation, Cl^-^ exit and thus cellular loss of KCl with osmotically obliged water [9].

The observed indoxyl sulfate induced cell membrane scrambling was similarly due to increased cytosolic Ca^2+^ activity. Accordingly, the presence of extracellular Ca^2+^ is required for full stimulation of cell membrane scrambling. The indoxyl sulfate concentrations required to trigger phosphatidylserine exposure were lower than those required to significantly enhance Fluo3 fluorescence and to decrease forward scatter. It must be kept in mind that Fluo3 fluorescence and forward scatter may be less sensitive than detection of annexin V binding erythrocytes. Thus, the present observations do not allow the conclusion that Ca^2+^ independent stimulation of cell membrane scrambling does occur at low indoxyl sulfate concentrations. Nevertheless, additional mechanisms may conctribute to the stimulation of cell membrane scrambling following indoxyl sulfate exposure. As shown here, indoxyl sulfate stimulates the formation of ceramide, which is in turn known to sensitize erythrocytes for the scrambling effects of increased cytosolic Ca^2+^ concentration [9]. Indoxyl sulfate induces oxidative stress [42,43], a well known trigger of eryptosis [9,12]. Indoxyl sulfate further activates p38 MAP kinase [47], which again has been shown to trigger eryptosis [16]. Moreover, the machinery governing eryptosis includes caspases [9,11,12], protein kinase C [9], AMP activated kinase AMPK [9], cGMP-dependent protein kinase [9], Janus-activated kinase JAK3 [13] and casein kinase [14,15]. At least in theory, those mechanisms may participate in the triggering of eryptosis by indoxyl sulfate.

Indoxyl sulfate is an uremic toxin, which could well contribute to the accelerated erythrocyte death in end stage renal disease. Phosphatidylserine exposing erythrocytes are bound to phagocytosing cells and are thus rapidly cleared from circulating blood [9]. In end stage renal disease, the accelerated loss of erythrocytes is paralleled by impaired formation of new erythrocytes thus leading to development of anemia [50]. The effect of indoxyl sulfate could be shared by other uremic toxins, which could similarly trigger eryptosis. Uremic toxins already known to trigger eryptosis include vanadate [9], acrolein [38] and methylglyoxal [9]. Moreover, eryptosis and thus clearance of affected erythrocytes from circulating blood is stimulated by iron deficiency [51], which is common in end stage renal disease and contributes to the development of anemia in those patients [5,6]. Clearly, additional substances or disorders could contribute to the development of anemia in patients with end stage renal disease.

Excessive eryptosis in end stage renal disease could further trigger thrombosis and impede microcirculation. Phosphatidylserine exposing erythrocytes adhere to the vascular wall at least in part by interaction of the erythrocyte phosphatidylserine and endothelial CXCL16/SR-PSO [52]. Erythrocyte adherence to the vascular wall is expected to interfere with blood flow [9,52]. Phosphatidylserine exposing erythrocytes may further stimulate blood clotting [9,53,54]. The curtailing of blood flow following enhanced turnover of erythrocytes with increased numbers of phosphatidylserine exposing erythrocytes in circulating blood may contribute to the side effects following uncritical use of erythropoietin or other erythropoiesis stimulating agents [55-57].

## Conclusion

The uremic toxin indoxyl sulfate triggers cell shrinkage and cell membrane scrambling and thus eryptosis, the suicidal death of erythrocytes. Indoxyl sulfate is at least partially effective by increasing cytosolic Ca^2+^ and ceramide formation.

## Competing interest

All authors of this manuscript declare that they have no competing interests.

## Authors’ contributions

MSEA and MA performed experiments, JV designed experiments and evaluated data, FL drafted the manuscript. All authors read and approved the final manuscript.

## Pre-publication history

The pre-publication history for this paper can be accessed here:

http://www.biomedcentral.com/1471-2369/14/244/prepub
